# Assessment of hepatitis C virus infection in two adjacent Thai provinces with drastically different seroprevalence

**DOI:** 10.1371/journal.pone.0177022

**Published:** 2017-05-05

**Authors:** Rujipat Wasitthankasem, Preeyaporn Vichaiwattana, Nipaporn Siripon, Nawarat Posuwan, Chompoonut Auphimai, Sirapa Klinfueng, Napha Thaneskongtong, Viboonsak Vuthitanachot, Supapith Saiyatha, Chaiwat Thongmai, Sarawut Suwanpatoomlerd, Saowakon Sochoo, Natnada Pongsuwan, Kittiyod Poovorawan, Pisit Tangkijvanich, Sompong Vongpunsawad, Yong Poovorawan

**Affiliations:** 1 Center of Excellence in Clinical Virology, Faculty of Medicine, Chulalongkorn University, Bangkok, Thailand; 2 Chumpae Hospital, Chum Phae, Khon Kaen, Thailand; 3 Phetchabun Provincial Public Health Office, Mueang Phetchabun, Phetchabun, Thailand; 4 Lomkao Crown Prince Hospital, Na-saeng, Lom Kao, Phetchabun, Thailand; 5 Department of Clinical Tropical Medicine, Faculty of Tropical Medicine, Mahidol University, Bangkok, Thailand; 6 Research Unit of Hepatitis and Liver Cancer, Department of Biochemistry, Faculty of Medicine, Chulalongkorn University, Bangkok, Thailand; University of Cincinnati College of Medicine, UNITED STATES

## Abstract

Improved awareness of the hepatitis C virus (HCV) transmission has contributed to the overall decline in the HCV infection rate in some developing countries including Thailand. Chronic HCV infection in some rural Thai communities, however, presents a challenge in the efforts to treat and manage HCV-related diseases. Published and unpublished studies have suggested an unusually high incidence of HCV infection in a Thai province of Phetchabun compared to elsewhere in Thailand. To determine the magnitude of HCV infection and identify potential factors contributing to the higher rate of HCV infection in this province, we performed a population-based study in Phetchabun (n = 1667) and the neighboring Khon Kaen province (n = 1410) where HCV prevalence is much lower. Individuals between 30 and 64 years old completed detailed questionnaires designed to identify HCV risk factors and provided blood samples for anti-HCV antibody screening. The anti-HCV seropositive rates were 15.5% (259/1667) in Phetchabun and 3.6% (51/1410) in Khon Kaen. Positive samples were subsequently genotyped for HCV core gene sequence and assessed for the hepatitis B virus surface antigen (HBsAg) and human immunodeficiency virus antigen/antibody (HIV Ag/Ab). More individuals in Phetchabun possessed the combined presence of HBsAg (5.0%) and HIV Ag/Ab (0.4%) than those in Khon Kaen (3.9% HBsAg and 0.0% HIV Ag/Ab). While male gender, intravenous drug use (IVDU) and tattoos were significant HCV risk factors in both provinces (p <0.05), education less than high school and agriculture-related occupation were additionally associated with HCV in Phetchabun. HCV genotypes 6, 3, and 1 were identified in similar frequency in both provinces. We estimated that prevalence of HCV seropositivity and viremic carriers were higher in Phetchabun (143 and 111 per 1000) than in Khon Kaen (34 and 22 per 1000). Finally, we derived a simple risk factor-based scoring system as a useful preclinical tool to screen individuals at risk of chronic HCV infection prior to intervention. Knowledge gained from this study will assist in HCV screening and promote access to anti-viral treatment in high-risk groups.

## Introduction

Hepatitis C virus (HCV) is a major cause of chronic liver disease, cirrhosis and hepatocellular carcinoma (HCC) [1,2] and affects approximately 185 million people worldwide [3]. HCV was often acquired from blood transfusion, iatrogenic procedure, intravenous drug use (IVDU), accidental needle sticks, unsterile needle use in medical procedures, and tattooing in the years before HCV pathogenesis was elucidated [4–6]. The presence of anti-HCV antibodies can indicate current or past HCV infection, and when left untreated, chronic infection can be as high as 75% to 85% [4,7].

The prevalence rates of HCV in developing countries are generally higher than in industrialized nations, but improving socio-economic status and education in developing nations have contributed in the decline in new HCV infection. For example, the overall HCV seroprevalence in Thailand has decreased from 2.2% to 0.9% within the past 10 years [8,9] and will likely be ≤0.2% over the next 20 years [10]. Despite the declining trend in the general population, HCV infection rate continues to increase among individuals >30 years with the highest prevalence among individuals 41–50 years [9]. Regional pockets of relatively high HCV endemicity also remained in northern and northeastern Thailand [9,11–13].

In 2006, The Bureau of Epidemiology of the Thai Ministry of Health reported a marked increase of HCV infection compared to 2004 [14]. Very limited seroprevalence survey in a rural province of Phetchabun found that up to 16% of the residents possessed HCV antibodies, well above the national average of 2.2% [14]. Although most individuals were asymptomatic (55.5%), chronic hepatitis (17.8%), cirrhosis (8.9%) and HCC (2.2%) were also reported [8,15]. However, accurate number of infection and contributing factors attributed to the high rate of HCV was unknown.

The apparently high rate of HCV infection in Phetchabun has not been independently confirmed. Since clinical development of HCV infection is slow and asymptomatic phase is long, we hypothesized that HCV prevalence in Phetchabun is significantly higher than initially assessed. Therefore, we sought to more accurately determine HCV seroprevalence rate and identify potential risk factors contributing to active infection in this province as compared to the neighboring province of Khon Kaen where HCV infection was low. We also proposed the use of a simple scoring system defined by several associated risk factors, which can be used to estimate crude HCV infection rate for a given community.

## Material and methods

**Fig 1:**
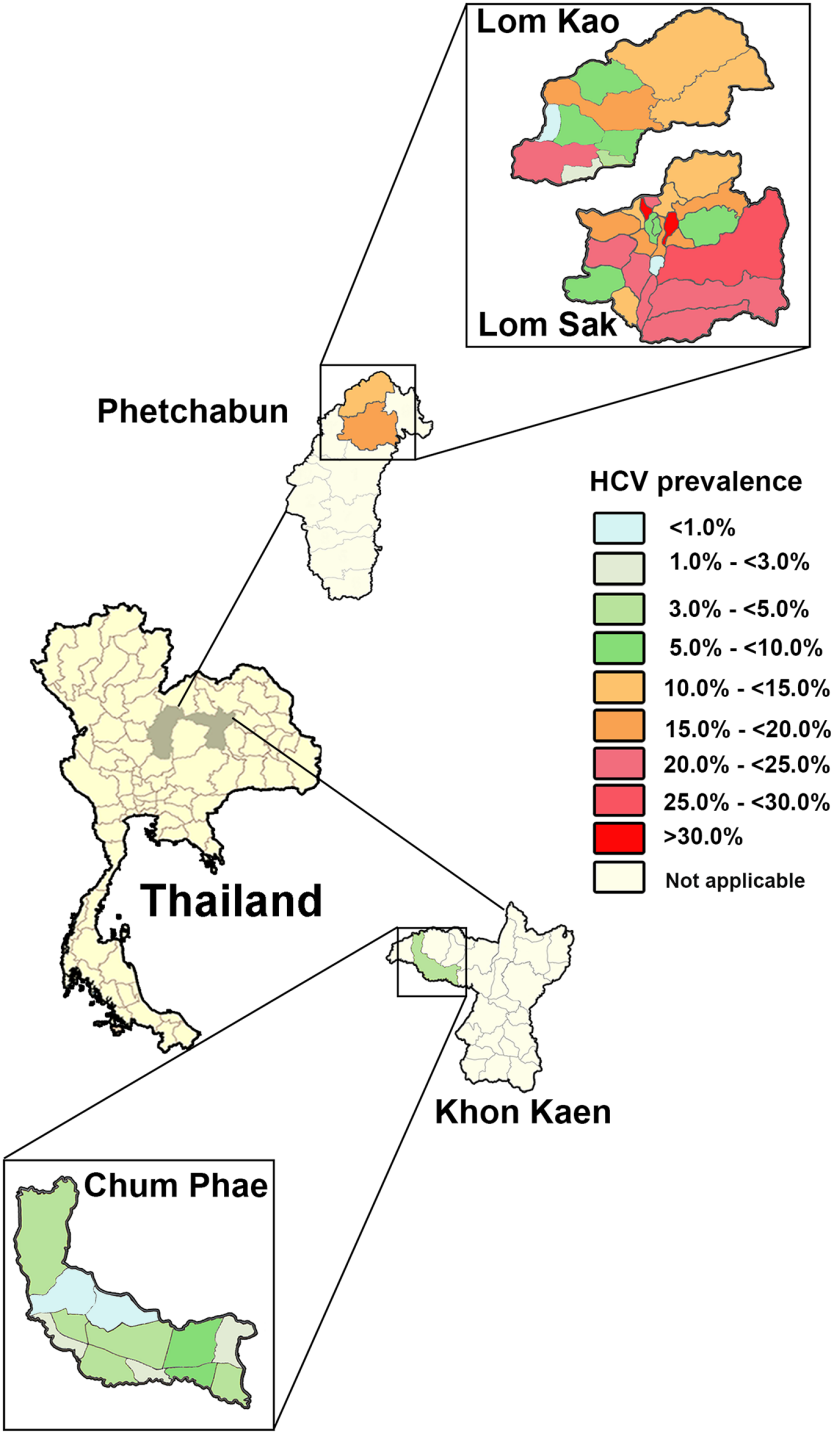
Geographical locations of the study sites. Phetchabun and Khon Kaen are adjacent provinces with different HCV prevalence. The districts of Lom Kao and Lom Sak in Phetchabun and Chum Phae in Khon Kaen are divided into administrative sub-districts. Sub-districts are shown in different colors in which light blue denotes relatively low (<1.0%) and red denotes relatively high (>30%) HCV seroprevalence. Regions with no data are denoted white.

The cohorts in this cross-sectional study reside in Phetchabun and Khon Kaen provinces of Thailand (Fig 1). Khon Kaen was a comparison site because it adjoins Phetchabun and previous serological data from the 2014 national survey of hepatitis burden were available [9]. Written informed consent was obtained from all participants and the study protocol was approved by the institutional review board of the Faculty of Medicine, Chulalongkorn University (IRB No. 258/58).

### Study population

**Fig 2:**
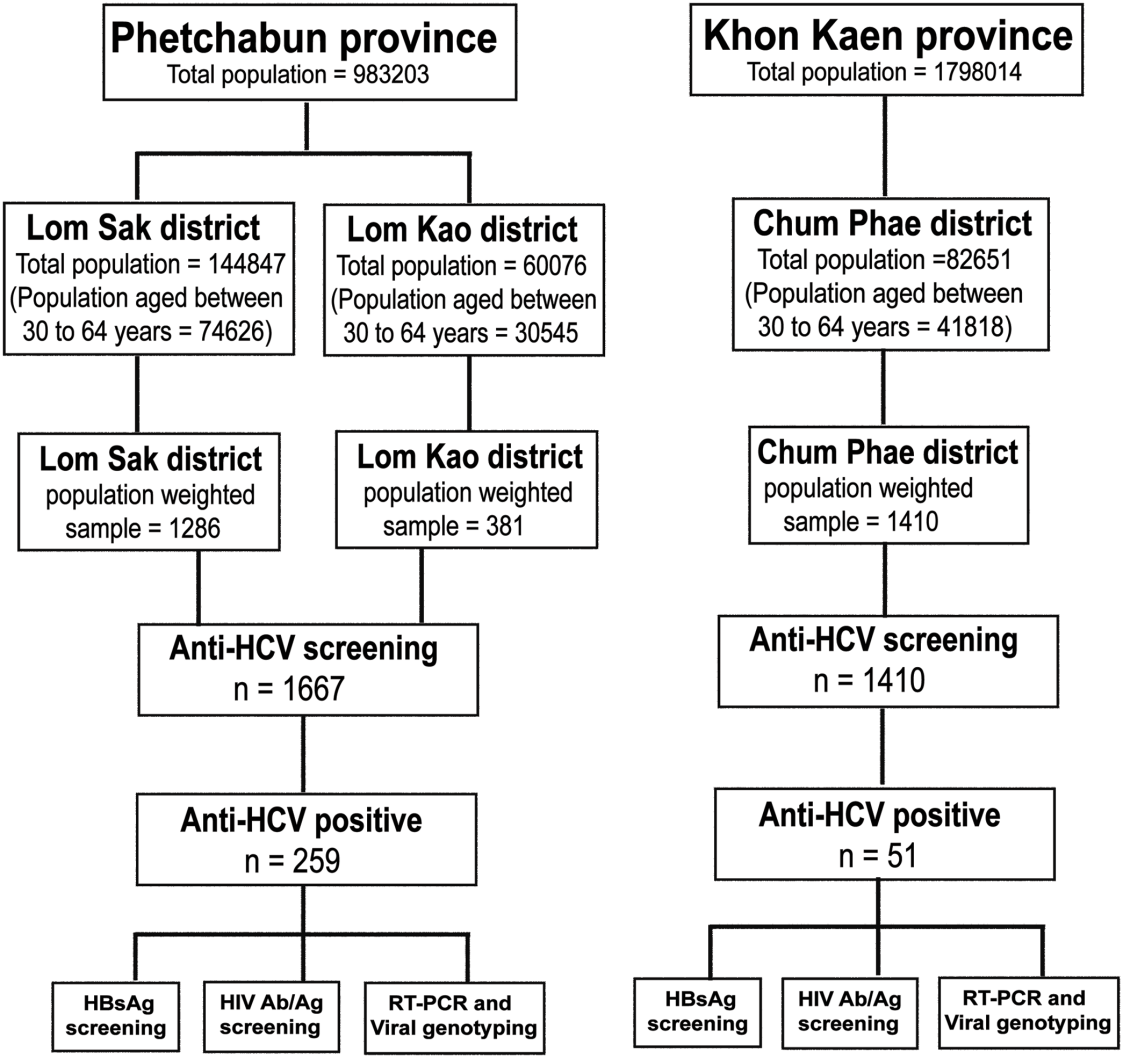
Schematic diagram of this HCV study. Data obtained from individuals between 30 to 64 years residing in Lom Sak and Lom Kao in Phetchabun and Chum Phae in Khon Kaen were analyzed. Samples tested positive for anti-HCV antibodies were further evaluated for HBV and HIV co-infection. HCV RNA was isolated and the virus genotyped by RT-PCR, sequencing, and phylogenetic analysis.

Randomly chosen individuals aged 30 to 64 years from Lom Kao and Lom Sak districts of Phetchabun (n = 1667) and Chum Phae district of Khon Kaen (n = 1410) were recruited between October and December 2015 (Fig 2). This age range was examined because they were more likely to be seropositive compared to younger individuals [9]. Individuals were generally in good health and were seen at Lom Kao Crown Prince Hospital (Phetchabun) and Chumpae Hospital (Khon Kaen) where they had their blood drawn. They were age-weighed according to the proportion of the population in each sub-district of Lom Kao, Lom Sak, and Chum Phae. Socio-demographic data and exposure to potential risk factors associated with HCV infection were obtained from completed questionnaires (e.g. history of blood transfusion, non-intravenous illicit drug use, IVDU, history of medical surgery, sharp needle/injection administered by licensed medical person or unlicensed non-medical person, tattooing, sharing of razors, acupuncture treatment, accidental needle stick, sexual orientation, having HCV-infected spouse, history of liver disease, and hepatitis disease in family member) (S1 and S2 Files).

### Serological and molecular assays

Anti-HCV antibody was evaluated using an automated chemiluminescent microparticle immunoassay (ARCHITECT anti-HCV assay, Abbott Diagnostics, Wiesbaden, Germany). Positive samples were tested for co-infection with HBV and/or HIV by automated HBsAg and HIV Ag/Ab assays, also using the ARCHITECT platform. For HCV genotyping, viral nucleic acid was extracted from the sera and nested RT-PCR performed to amplify the HCV partial core region as previously described [16]. Primers used for the first-round of PCR were 954F (ACTGCCTGATAGGGTGCTTGCGAG, nucleotide position 288–311 based on the reference strain with GenBank accession number M62321) and 410R (ATGTACCCCATGAGGTCGGC, position 732–751). Primers for the second-round of PCR were 953F (AGGTCTCGTAGACCGTGCATCATG, position 321–344) and 951R (CACTGTRAGGGTATCGATGAC, position 705–725) [17]. The expected 405 base-pair amplicons were resolved by 2% agarose gel electrophoresis and subjected to nucleotide sequencing. HCV sequences were preliminarily assigned genotypes using BLASTN (http://www.ncbi.nlm.nih.gov) and viral genotyping tool (http://www.ncbi.nlm.nih.gov/projects/genotyping/formpage.cgi).

### Phylogenetic analysis of HCV genotypes

Confirmation of HCV genotypes was performed using phylogenetic analysis. Nucleotide sequences were edited and assembled using BioEdit version.5.0.9 and SeqMan versoin.8.1.3 and aligned with reference strains using ClustalX version 2.1. Phylogenetic tree was constructed using Kimura two-parameter best-fit model and 1,000 bootstrap value implemented in MEGA software version 6. Nucleotide sequences obtained from this study were deposited in GenBank (accession numbers KY625212 to KY625445). Reference sequences used in this study were 1a (M62321, M67463, EU155350), 1b (D90208, M58335, AF207757, EU155303), 2a (AB047639, D00944), 2b (D10988, AB030907), 2c (D50409), 3a (D17763, D28917, AB691595, KJ470615-), 3b (D49374), 4a (Y11604), 5a (Y13184), 6a (Y12083, AY859526), 6b (D84262), 6c (EF424629), 6d (D84263), 6e (DQ314805), 6f (DQ835760, EU246936, HM042030), 6g (D63822), 6h (D84265), 6i (DQ835770), 6j (DQ835769), 6k (D84264), 6l (EF424628), 6m (DQ835767), 6n (DQ278894, DQ835768), 6o (EF424627), 6p (EF424626), 6q (EF424625), 6r (EU408328), 6s (EU408329), 6t (EF632071, EU246939), 6u (EU246940), 6v (EU158186, EU798760), 6w (DQ278892, EU643834), 6xa (EU408330, EU408332), 6xb (JX183552, KJ567645), 6xc (KJ567650, KJ567649), 6xd (KM252791, KM252789), 6xe (JX183557, KM252792) and 7a (EF108306).

### Estimating the HCV carrier rate

Population census data of Lom Kao, Lom Sak, and Chum Phae in 2015 were obtained from the Official Statistics Registration System (http://stat.dopa.go.th/stat/statnew/upstat_age.php). HCV seropositive rates were age group-stratified (from 30 to 64 years). Seropositive carrier rates were extrapolated and calculated separately for men and women.

### Statistical analysis

All categorical data were transformed to nominal scale. Comparison of the differences in potential risk factors for HCV infection between groups was done using Chi-square and ANOVA univariate analysis (IBM SPSS Statistics version 22, IBM Corporation, Armonk, NY). A *p*-value <0.05 was considered statistically significant. Factors with a *p*-value <0.2 in the univariate analysis were further evaluated for independent effect using multivariate analysis with logistic regression model. Odds ratios and 95% confidence interval (CI) of demographic and relative risk factors were derived from univariate and multivariate analyses. The odds ratios of significant risk factors were used to develop the screening score for HCV infection prediction. Receiving operating curve (ROC) analysis was used to calculate the area under the curve (AUC) in order to interpret validity of screening test for HCV infection. Cut-off score with sensitivity (≥ 80%) were chosen to maximize the inclusion of asymptomatic HCV-positive individuals.

## Results

### Prevalence and factors associated with HCV seropositivity

The mean age of the entire study population (n = 3077) was 48.4 ± 7.9 years and women comprised 56.8% (1747/3077) (Table 1). The majority of individuals (60.2%, 1831/3041) possessed at least some primary school education (grades 1 through 6). The most common occupation was agriculture (2144/2996, 71.6%). Individuals who tested positive for HCV antibody were generally older, less educated, and overwhelmingly male.

**Table 1:** Demographic data of individuals in Phetchabun and Khon Kaen in this HCV seroprevalence study.

Demographic variables	Total (N = 3077)			Phetchabun (N = 1667)			Khon Kaen (N = 1410)		
	All	Anti HCV positive (%)	*P* value	All	Anti HCV positive (%)	*P* value	All	Anti HCV positive (%)	*P* value
**Mean age (±SD)**	48.4 (±7.9)	49.4 (±7.3)	0.011^a^	47.9 (±7.8)	49.2 (±7.3)	0.003^a^	48.9 (±7.8)	50.4 (±7.0)	0.159
**Age group (years)**			0.113			0.058			0.439
30–39	462	34 (7.4)		270	31 (11.5)		192	3 (1.6)	
40–49	1170	114 (9.7)		652	94 (14.4)		518	20 (3.9)	
50–59	1210	136 (11.2)		633	113 (17.8)		577	23 (4.0)	
60–64	235	26 (11.1)		112	21 (18.8)		123	5 (4.1)	
TOTAL	3077	310 (10.1)		1667	259 (15.5)		1410	51 (3.6)	
**Gender**			<0.001^a^			<0.001^a^			<0.001^a^
Male	1330	262 (19.7)		774	220 (28.4)		556	42 (7.5)	
Female	1747	48 (2.7)		893	39 (4.4)		854	9 (1.0)	
**Education**			0.001^a^			<0.001^a^			0.879
No formal education	3	1 (33.3)		2	1 (50.0)		1	0 (0.0)	
Grade 1–6	1831	199 (10.9)		945	169 (17.9)		886	30 (3.4)	
Grade 7–9	480	57 (11.9)		270	47 (17.4)		210	10 (4.8)	
Grade 10–12	538	45 (8.4)		309	36 (11.6)		229	9 (3.9)	
University or higher	189	4 (2.1)		115	2 (1.7)		74	2 (2.7)	
Total	3041	306 (10.1)		1641	255 (15.5)		1400	51 (3.6)	
**Occupation**			0.004^a^			<0.001^a^			0.199
Agriculture	2144	242 (11.3)		1111	204 (18.4)		1033	38 (3.7)	
Temporary employee	475	41 (8.6)		286	36 (12.6)		189	5 (2.6)	
Business owner	78	4 (5.1)		55	4 (7.3)		23	0 (0.0)	
Government employee/ officer	75	3 (4.0)		39	2 (5.1)		36	1 (2.8)	
State enterprise employee	3	0 (0.0)		1	0 (0.0)		2	0 (0.0)	
Clinic/hospital worker	13	0 (0.0)		11	0 (0.0)		2	0 (0.0)	
Monkhood	31	4 (12.9)		0	0 (0.0)		31	4 (12.9)	
Others	177	6 (3.4)		124	5 (4.0)		53	1 (1.9)	
Total	2996	300 (10.0)		1627	251 (15.4)		1369	49 (3.6)	

^a^ denoted statistical significance

% calculated according to each characteristic

Lom Kao and Lom Sak are adjacent districts approximately 40 kilometers apart, while Chum Phae is 120 kilometers east of Lom Sak. The differences in the HCV seropositivity rates in these districts, however, were striking (Fig 1). In both provincial cohorts, individuals were similar in age, gender ratio, education level, and occupation. Although the overall HCV seroprevalence was 10.1%, the rate was significantly higher in Phetchabun (Lom Sak = 11.8%, Lom Kao = 16.6%, total = 15.5%) than in Khon Kaen (Chum Phae = 3.6%) (Table 1). The proportion of seropositive men compared to seropositive women were approximately 7:1 in both provinces (28.4% vs. 4.4% in Phetchabun, *p*<0.001; 7.5% vs. 1.0% in Khon Kaen, *p*<0.001). Several factors associated with HCV seropositivity were significantly linked to the Phetchabun but not Khon Kaen cohort. These were the slightly higher mean age (*p* = 0.003), education (*p*<0.001) and agricultural employment (*p*<0.001).

In the Phetchabun cohort, univariate analysis showed several factors were associated with increasing odds ratios of HCV seropositivity (Table 2 and S1 Table). These included age, male gender, education less than university level, agricultural and temporary employment, history of oral drug abuse and IVDU. HCV-seropositive individuals were also more likely to have a history of medical treatment involving injection either by medical or non-medical staff, shared razor blade, and had tattoos. Lower HCV seropositivity rates were observed in individuals with a history of surgery (OR 0.6, 95% CI: 0.5, 0.8) and hepatitis in the family (OR 0.6, 95% CI: 0.4, 1.0). In Khon Kaen, however, higher HCV seropositivity rates were statistically significant in men, oral drug abuser, IVDU and those with tattoo.

**Table 2:** Univariate analysis of factors associated with HCV infection in Phetchabun and Khon Kaen.

Parameters	Phetchabun				Khon Kaen			
	All	Anti-HCV positive (%)	Unadjusted		All	Anti-HCV positive (%)	Unadjusted	
			odds ratio (95% CI)	*P* value			odds ratio (95% CI)	*P* value
**Age**^a^	1667	49.2 (7.3)	1.3 (1.1, 1.5)	0.003^b^	1410	50.4 (7.03)	1.3 (0.9, 1.9)	0.160
**Male/Female**	774/893	220 (28.4)/39 (4.4)	8.7 (6.1, 12.4)	<0.001^b^	551/854	42 (7.6)/9 (1.0)	7.7 (3.7, 15.9)	<0.001^b^
**Education**	1641				1400			
≤ Grade 6	947	170 (17.9)	12.4 (3.0, 50.5)	<0.001^b^	887	30 (3.4)	1.3 (0.3, 5.4)	0.755
Grade 7–9	270	47 (17.4)	11.9 (2.8, 49.9)	0.001^b^	210	10 (4.8)	1.8 (0.4, 8.4)	0.455
Grade 10–12	309	36 (11.6)	7.4 (1.8, 31.5)	0.006^b^	229	9 (3.9)	1.5 (0.3, 7.0)	0.626
University or higher	115	2 (1.7)	1 (-, -)		74	2 (2.7)	1 (-, -)	
**Occupation**	1627				1369			
Agriculture	1111	204 (18.4)	5.3 (2.2, 13.3)	<0.001^b^	1033	38 (3.7)	2.0 (0.3, 14.8)	0.502
Temporary employee	286	36 (12.6)	3.4 (1.3, 9.0)	0.012^b^	189	5 (2.6)	1.4 (0.2, 12.4)	0.755
Business owner	55	4 (7.3)	1.9 (0.5, 7.2)	0.367	23	0 (0.0)	N/A	N/A
Government employee	39	2 (5.1)	1.3 (0.8, 0.2)	0.769	36	1 (2.8)	1.5 (0.1, 24.5)	0.782
Monkhood	0	0 (0)	N/A	N/A	31	4 (12.9)	7.7 (0.8, 72.4)	0.074
Others	136	5 (3.7)	1 (-, -)		57	1 (1.7)	1 (-, -)	
**Oral drug abuse**	1635		6.5 (4.1, 10.3)	<0.001^b^	1405		3.6 (1.0, 12.5)	0.042^b^
No	1554	211 (13.6)			1379	48 (3.5)		
Yes	81	41 (50.6)			26	3 (11.5)		
**Intravenous drug use**	1635		23.8 (11.6, 48.5)	<0.001^b^	1398		146.3 (16.7, 1277.6)	<0.001^b^
No	1588	214 (13.5)			1392	46 (3.3)		
Yes	47	37 (78.7)			6	5 (83.3)		
**History of surgery**	1647		0.6 (0.5, 0.8)	0.003^b^	1407		0.8 (0.4, 1.5)	0.467
No	1073	186 (17.3)			888	34 (3.8)		
Yes	574	67 (11.7)			519	16 (3.1)		
**Injection by medical staff**	1602		1.4 (1.0, 1.8)	0.022^b^	1392		1.2 (0.6, 2.5)	0.649
No	853	118 (13.8)			1169	40 (3.4)		
Yes	749	135 (18.0)			223	9 (4.0)		
**Injection by non-medical staff**	1612		1.7 (1.2, 2.3)	0.002^b^	1395		1.4 (0.6, 3.4)	0.446
No	1362	196 (14.4)			1270	44 (3.5)		
Yes	250	55 (22.0)			125	6 (4.8)		
**Sharing razor blade**	1641		2.0 (1.4, 2.8)	<0.001^b^	1402		1.4 (0.6, 3.1)	0.454
No	1451	208 (14.3)			1248	42 (3.4)		
Yes	190	47 (24.7)			154	7 (4.5)		
**Tattooing**	1641		4.3 (3.2, 5.7)	<0.001^b^	1395		3.7 (2.0, 6.8)	<0.001^b^
No	1343	150 (11.2)			1213	33 (2.7)		
Yes	298	104 (34.9)			182	17 (9.3)		
**Hepatitis within family**	1635		0.6 (0.4, 1.0)	0.033^b^	1355		0.4 (0.1, 1.9)	0.266
No	1386	224 (16.2)			1239	47 (3.8)		
Yes	249	27 (10.8)			116	2 (1.7)		

^a^Age, data presented as mean (SD), age 10-year interval was use in statistical analysis.

^b^ denoted statistical significant

N/A; not applicable due to a small cell count

% calculated according to each characteristic

Multivariate analysis by logistic regression was performed on 12 parameters for Phetchabun and 4 parameters for Khon Kaen (Table 3). In the Phetchabun cohort, independent factors associated with HCV seropositive were male gender (adjusted OR 5.7, 95% CI: 3.7, 8.7), education between grade 7 to 9 (adjusted OR 9.6, 95% CI: 1.2, 76.1), agricultural occupation (adjusted OR 2.9, 95% CI: 1.1, 7.8), IVDU (adjusted OR 14.4, 95% CI: 5.3, 38.6), and having tattoo (adjusted OR 2.2, 95% CI: 1.5, 3.1) after adjusted for age (10 years interval), history with oral drug abuse, received operation, therapeutic injection by medical and non-medical staff, sharing razor blade and having hepatitis disease in family member. In the Khon Kaen cohort, after adjusted for history with oral drug abuse, the significant risk factors were male gender (adjusted OR 6.0, 95% CI: 2.8, 12.8), IVDU (adjusted OR 73.7, 95% CI: 5.1, 1074.1) and having tattoo (adjusted OR 2.0, 95% CI: 1.1, 3.9).

**Table 3:** Multivariate analysis of factors associated with HCV infection in Phetchabun and Khon Kaen.

	Phetchabun		Khon Kaen	
Parameters	Adjusted		Adjusted	
	odds ratio (95% CI)	*P* value	odds ratio (95% CI)	*P* value
**Age**^a^	1.1 (0.9, 1.4)	0.419	-	-
**Male/Female**	5.7 (3.7, 8.7)	<0.001^b^	6.0 (2.8, 12.8)	<0.001^b^
**Education**				
≤ Grade 6	7.5 (1.0, 58.6)	0.054	-	-
Grade 7–9	9.6 (1.2, 76.1)	0.032^b^	-	-
Grade 10–12	6.8 (0.9, 53.6)	0.070	-	-
University or higher	1 (-, -)		-	-
**Occupation**				
Agriculture	2.9 (1.1, 7.8)	0.038^b^	-	-
Temporary employee	2.1 (0.7, 6.2)	0.168	-	-
Others	1 (-, -)		-	-
**Oral drug abuse**	0.9 (0.5, 1.9)	0.896	0.4 (0.0, 3.4)	0.405
**Intravenous drug use**	14.4 (5.3, 38.6)	<0.001^b^	73.7 (5.1, 1074.1)	0.002^b^
**History of surgery**	1.1 (0.8, 1.7)	0.461	-	-
**Injection by medical staff**	1.1 (0.8, 1.5)	0.631	-	-
**Injection by non-medical staff**	1.2 (0.8, 1.9)	0.306	-	-
**Sharing razor blade**	1.1 (0.7, 1.8)	0.759	-	-
**Tattooing**	2.2 (1.5, 3.1)	<0.001^b^	2.0 (1.1, 3.9)	0.034^b^
**Hepatitis within family**	0.8 (0.5, 1.4)	0.453	-	-

^a^Age, age 10-year interval was use in statistical analysis.

^b^ denoted statistical significant

**Fig 3:**
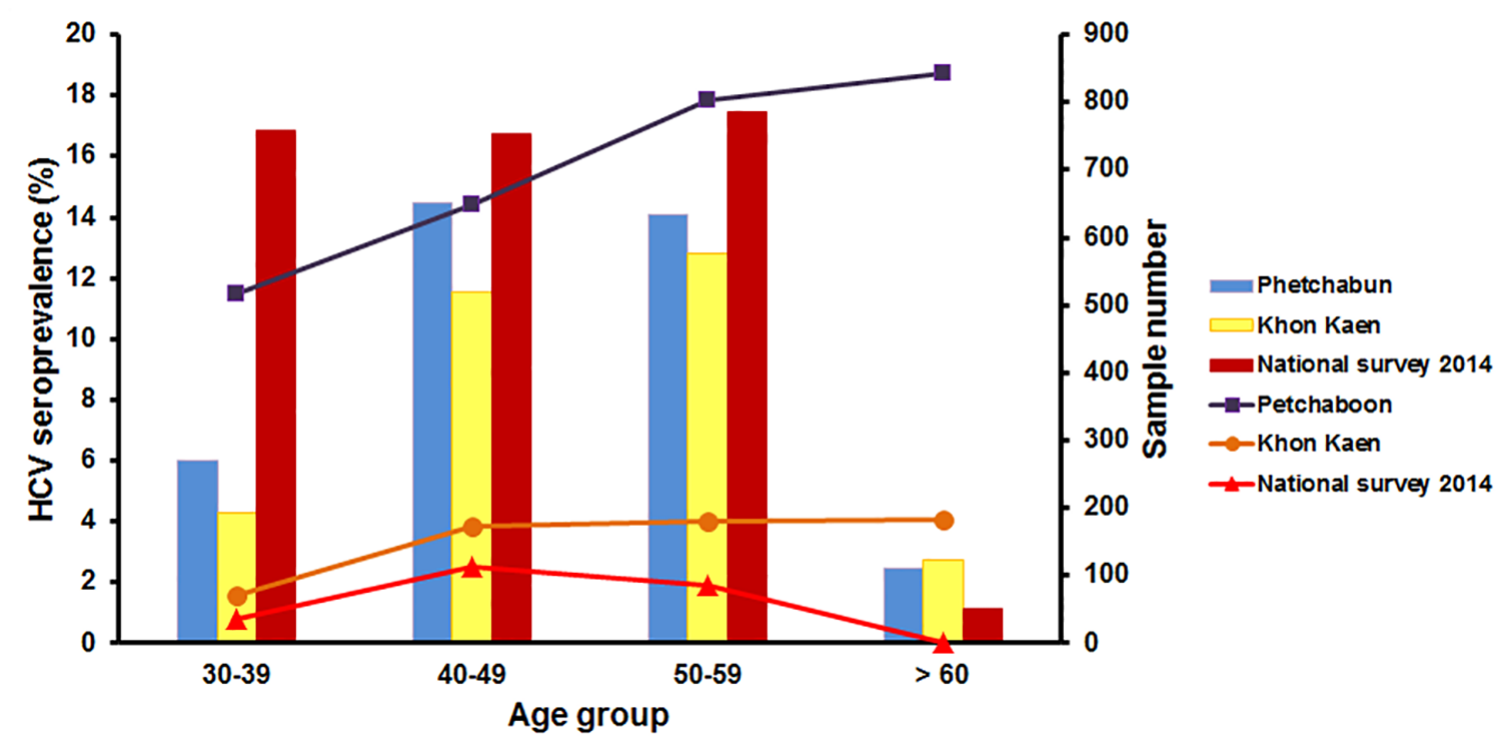
Anti-HCV seroprevalence in Phetchabun and Khon Kaen in this study compared to data from the national survey in 2014. HCV seroprevalence is indicated by the line graphs (left scale). The number of individuals according to age groups included in the study is represented by the bar graphs (right scale).

The 15.5% HCV seropositivity in Phetchabun from this study was 4 times higher than the rate in the adjacent province of Khon Kaen (Fig 3). In addition, seroprevalence in Phetchabun was consistently higher than that of Khon Kaen among all age groups and was 16.5 times higher than the national average.

### HCV co-infection with HBV or HIV

We assessed the prevalence of two additional bloodborne viruses, HBV and HIV, in HCV seropositive samples. HBsAg was found reactive in 13/259 (5.0%, male/female = 9/4) of the Phetchabun cohort and 2/51 (3.9%, male/female = 2/0) in the Khon Kaen cohort. From 266 individuals who provided consent for HIV Ag/Ab testing, only one individual in the Phetchabun cohort tested reactive for HIV Ag/Ab (1/228).

### Determination of HCV genotype

HCV RNA was found in 203 (78.4%) and 31 (60.8%) of the seropositive samples from Phetchabun and Khon Kaen, respectively (Table 4 and S1 Fig). Nucleotide sequence analysis showed that genotypes 3a and 6f were most commonly found in both provinces, while genotype 6c was present in only one sample. In Phetchabun where there were more HCV-positive samples, genotype 1a comprised a significant proportion of HCV identified. In Khon Kaen, genotype 1b was also frequently found. Overall, there was no statistically significant association between HCV genotypes and the presence of HBsAg, HIV status, transmission routes, or risk factors. Among the 13 HBsAg-positive samples, HCV RNA was present in 6 samples [genotype 3a (n = 3), 6f (n = 2) and 6n (n = 1)]. One sample positive for HIV Ag/Ab had HCV genotype 6n.

**Table 4:** HCV genotypes relative to gender and age by province.

	Total			Phetchabun			Khon Kaen		
	N (%)	Sex (M/F)	Mean age (SD)	N (%)	Sex (M/F)	Mean age (SD)	N (%)	Sex M/F)	Mean age (SD)
**HCV seropositive**	310 (10.1)	262/48	49.43 (7.3)	259 (15.5)	220/39	49.24 (7.3)	51 (3.6)	42/9	50.39 (7.03)
**HCV RNA positive**	234 (75.5)	206/28	49.41(7.10)	203 (78.4)	180/23	49.36 (7.1)	31 (60.8)	26/5	49.68 (7.21)
**Genotype**									
1a	51 (21.8)	46/5	50.3 (7.3)	47 (23.2)	43/4	50.3 (7.3)	4 (12.9)	3/1	50.8 (7.4)
1b	23 (9.8)	23/0	49.3 (7.0)	17 (8.4)	17/0	48.9 (7.1)	6 (19.4)	6/0	50.5 (7.3)
3a	70 (29.9)	61/9	47.6 (7.4)	62 (30.5)	54/8	47.8 (7.4)	8 (25.8)	7/1	46.4 (8.4)
3b	3 (1.3)	3/0	45.3 (1.5)	2 (1.0)	2/0	46.0 (1.4)	1 (3.2)	1/0	44.0 (0.0)
6c	1 (0.4)	1/0	45.0 (0.0)	0 (0.00)	0/0	0.0 (0.0)	1 (3.2)	1/0	45.0 (0.0)
6f	71 (30.3)	60/11	51.3 (6.6)	64 (31.5)	54/10	51.1 (6.5)	7 (22.6)	6/1	53.9 (7.0)
6i	5 (2.1)	4/1	51.8 (2.9)	2 (1.0)	2/0	52.5 (3.5)	3 (9.7)	2/1	51.3 (3.1)
6n	10 (4.3)	8/2	44.1 (5.3)	9 (4.4)	8/1	44.2 (5.6)	1 (3.2)	0/1	43.0 (0.0)

### Estimates of HCV carriers

We next estimated the number of individuals with anti-HCV antibody, and HCV RNA carriers for each age group (between 30 and 64 years) and gender (S2 Table). In Phetchabun, anti-HCV antibody-positive individuals and HCV RNA carriers were estimated at 14261 per 100000 (male-to-female = 5.5:1) and 11061 per 100000 (male-to-female = 7.1:1), respectively. In Khon Kaen, HCV prevalence was estimated at 3424 per 100000 (male-to-female = 5.5:1) anti-HCV positive individuals and 2224 per 100000 (male-to-female = 6.9:1) viremic carriers.

### HCV infection screening score

**Fig 4:**
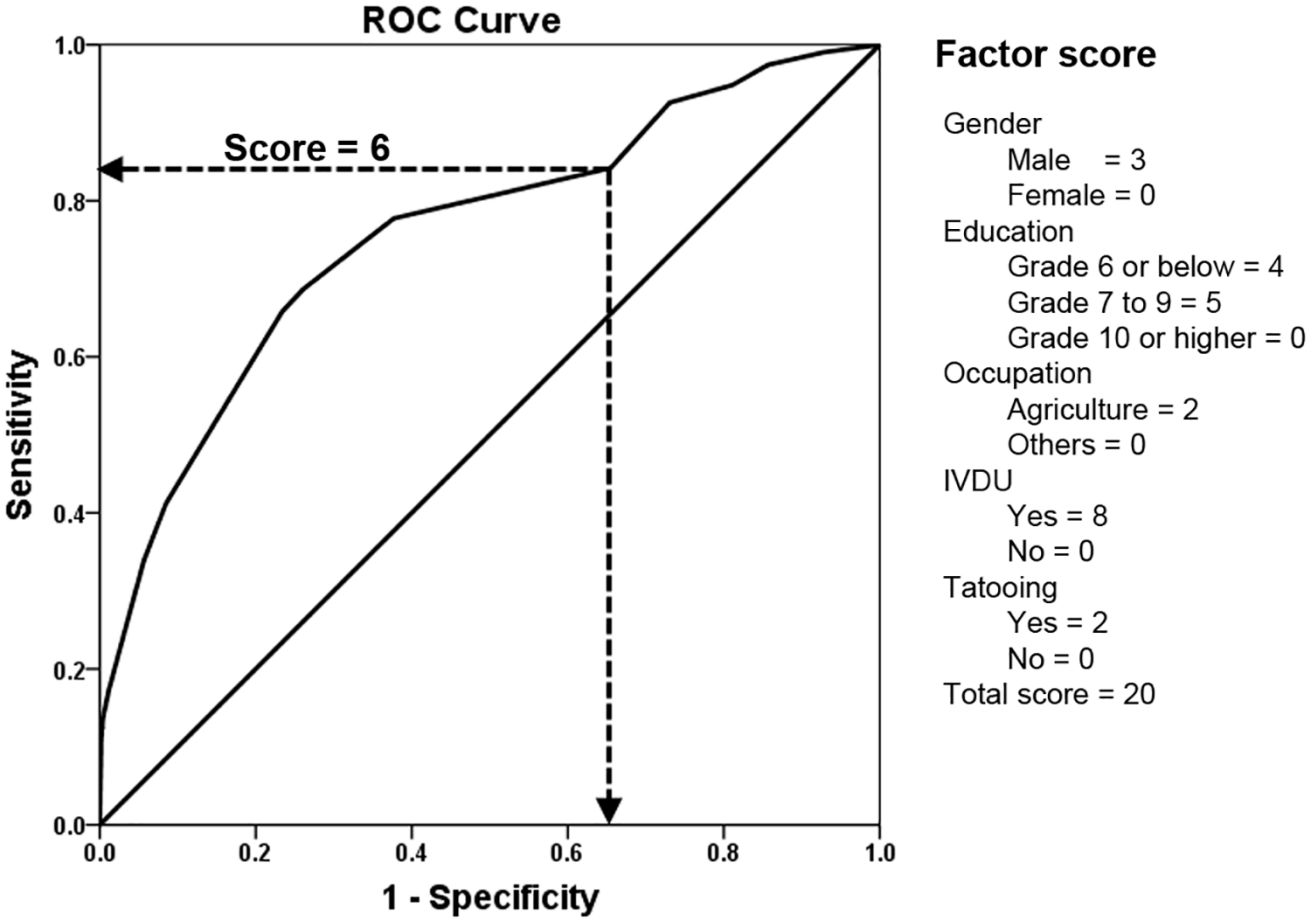
Receiving operating curve (ROC) of HCV screening score. The ROC represents an area under the curve (AUC) of HCV screening tool derived from factors associated with HCV infection. To maximize ascertainment of individuals with HCV, the cut-off score at 6 was chosen for the relatively high sensitivity at the expense of specificity.

To develop an algorithm for screening HCV in a given population, which can be used to ascertain HCV burden in the community prior to invasive diagnostics, statistically significant parameters in multivariate analysis were applied. Variables from Table 3 were selected and scored according to their odd ratios as follow: gender score = 3 for male and = 0 for female; education score = 4 for below grade 7, = 5 for grade 7–9 level, and = 0 for others; occupation score = 2 for agriculture and = 0 for others; IVDU score = 8 and = 0 for non-user; and tattooing score = 2 and = 0 for no tattoo. The sum scores of gender, education, occupation, IVDU and tattooing yield a total maximum risk score of 20. Validity of the risk screening score was analyzed by the receiving operating curve (ROC) and accuracy of the test was interpreted based on the area under the curve (AUC) (Fig 4). When applied to the data, AUC was 0.77 for Phetchabun (95% CI: 0.74, 0.80), 0.71 for Khon Kaen (95% CI: 0.62, 0.79) and 0.76 overall (95% CI: 0.73, 0.79). At the cut-off score ≥ 6.0, the sensitivity and specificity of the screening test of the overall data were 84.2% and 34.5%, respectively (Fig 4). Individuals who attained a cut-off score ≥ 6 by this screening test would therefore be advised to undergo further diagnostic test for HCV infection.

## Discussion

It was estimated that Thailand has a moderate HCV seroprevalence (1.7 to 2.3%) [3] and that 2.7% of Thais 15 years and older are HCV-seropositive [18]. A recent national study involving serosurvey of individuals residing in multiple provinces, however, revealed a more accurate and much lower overall HCV seroprevalence of 0.9% [9]. Nevertheless, there are regional variations in the past and present HCV exposure within Thailand. A small sero-survey in rural Phetchabun suggested that as high as 16% of the residents possessed HCV antibodies, which led us to examine the burden of HCV in a larger cohort in Phetchabun and in neighboring Khon Kaen where HCV data from previous studies only showed ~3.0% HCV seropositivity (S3 Table) [9,14].

The association of anti-HCV antibody with male gender, history of IVDU and tattooing is consistent with previous published reports [19–22]. In particular, IVDU is a major risk factor associated with HCV (adjusted OR 14.4 in Phetchabun and 73.7 in Khon Kaen) and is confirmed in several cohort studies [23,24]. Active infection as measured by the presence of serum HCV RNA was as high as 70% among IVDU compared to other modes of illicit drugs [25]. In fact, HCV seropositivity was a surrogate for the history of injected drug use in an HIV study [26]. Among HIV-positive young men, HCV co-infection was 20.0% in IVDU compared to 5.7% in non-IVDU [27].

Socioeconomic conditions such as education, occupation and income were reported as independent factors for HCV infection and indeed associated with HCV seropositivity in Phetchabun [28]. Among blood donors, HCV seropositivity was significantly associated with primary educational level [19]. In industrialized countries, education less than high school was identified as an independent risk factor for HCV infection in the United States [24], Denmark [28], France [22] and Italy [29]. Interestingly, agriculture-related occupation appears to be an independent risk factor for HCV in Thailand [19], although the specific behavior which led to the significant high prevalence of anti-HCV antibody in these individuals could not be ascertained from this study. It is possible that laborers comprising of mostly men with lower education often pursue agricultural work and have limited knowledge of the transmission of bloodborne pathogen, thus are at an increased risk of acquiring HCV.

HCV genotypes 1, 3, and 6 are known to be predominant in Thailand [9,16] and were the leading subtypes found in this study. The preponderance of subtype 6f in this region has fueled the speculation that this genotype originates in Southeast Asia [30–32]. The estimated number of HCV carriers derived from this study, if accurate, represents a significant burden of HCV and possible future cases of liver cirrhosis and hepatocellular carcinoma in Phetchabun. Treatment options with highly effective antivirals can promote viral clearance, but they are expensive and inaccessible for many individuals living in rural Thailand. Monitoring of clinical symptoms in actively infected individuals, prevention of transmission to family members and others in the community through public health interventions should therefore be a priority.

HBV and HIV, both of which are bloodborne with similar transmission routes and associated risk factors as HCV, were also examine. In this study, the HBV co-infection rate was 3.9% to 5.0% similar to the previously determined carrier rate (~4.5%) in the general Thai population of adults ≥ 27 year born before the implementation of the universal HBV immunization program [33]. In contrast to the relatively high prevalence of HCV, HIV burden was extremely low (only one instance in Phetchabun), suggesting that HCV dissemination in this region may have preceded the HIV epidemic that began around 3 decades ago.

There are many developing countries with similar socio-economic status, HCV seroprevalence, and challenges in HCV eradication as Thailand. To provide a tool for HCV risk assessment, which could be utilized in other world regions, we used the associated parameters linked to HCV seropositivity to develop a screening test. We found that the validity (AUC) of the test was 0.71 to 0.77. When the cut-off score was ≥ 6.0, the test showed 84.2% sensitivity and 34.5% specificity. We recommend this cut-off score as a preclinical screening threshold to include people who are at high risk of HCV infection prior to validating with laboratory diagnostics. Consequently, individuals who possess a cut-off score ≥ 6 should therefore avoid donating blood.

This study highlights the substantial burden of HCV infection in some rural areas of Thailand, but it has limitations. Information on HCV-associated clinical data such as liver enzyme functions, hepatic fibrosis, cirrhosis, and HCC were not sufficiently available for analysis. HCV seroprevalence was not determined for residents outside of the 30–64 year age range and living in other parts of Phetchabun. Since perhaps individuals with lower HCV risk were not included in this study, some parameters showed significance in univariate but not in multivariate analysis. Nevertheless, these data revealed the extent of HCV infection in Phetchabun, which may serve as a model for the Thai Ministry of Public Health and healthcare providers towards a better understanding of HCV epidemiology in rural Thailand. It is hoped that these data will foster a more effective public health policy and open up access to affordable treatment in endemic regions through universal health care coverage.

## Supporting information

S1 Fig Phylogenetic analysis of HCV core sequence of reference strains and those identified in this study. Region of 295 nucleotides in length were compared to the reference HCV sequences of genotypes 1 to 7 (bolded) using MEGA. Bootstrap values >80 are indicated at the nodes. The scale bar denote nucleotide difference between close relatives.
(TIF)

S1 File Questionaires (English lanquage). (PDF)

S2 File Questionaires (Thai language). (PDF)

S1 Table Unassociated factors in univariate analysis with HCV infection in Phetchabun and Khon Kaen. (DOCX)

S2 Table The estimated number of HCV-seropositive and actively infected individuals in the Lom Kao and Lom Sak districts in Phetchabun and in the Chum Phae district of Khon Kaen. (DOCX)

S3 Table HCV seroprevalence in Chum Phae district in Khon Kaen in 2014 [9] and this study. (DOCX)
