# Supplementary figures and images for: Assessment of hepatitis C virus infection in two adjacent Thai provinces with drastically different seroprevalence
Source: PLoS One. 2017 May 5;12(5):e0177022. doi: 10.1371/journal.pone.0177022 (PMC5419576; doi:10.1371/journal.pone.0177022)

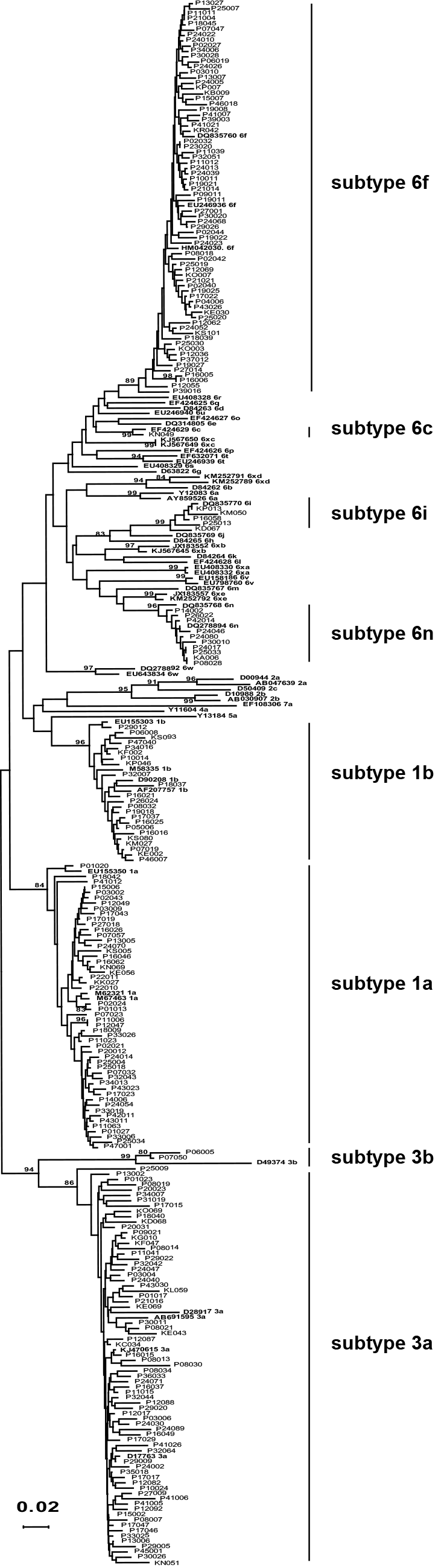

Supplement: S1 Fig — Region of 295 nucleotides in length were compared to the reference HCV sequences of genotypes 1 to 7 (bolded) using MEGA. Bootstrap values >80 are indicated at the nodes. The scale bar denote nucleotide difference between close relatives. (TIF) [file pone.0177022.s001.tif]
