# Supplementary material for: Assessment of hepatitis C virus infection in two adjacent Thai provinces with drastically different seroprevalence
Source: PLoS One. 2017 May 5;12(5):e0177022. doi: 10.1371/journal.pone.0177022 (PMC5419576; doi:10.1371/journal.pone.0177022)
Supplement: S1 File — (PDF) [file pone.0177022.s002.pdf]

Questionnaires (English language)

Code________________________ Information collected on Day___Month ______Year______

1. Age ☐☐ year Birth date Day/Month/Year ___/___/______

2. Gender ☐ 1. Male ☐ 2. Female

3. Weight _________kilogram 4. Height__________centimeters

5. Domicile

   Province__________________District_____________________Subdistrict_____________________

6. Educational level ☐ 1. Primary ☐ 2. Lower Secondary

   ☐ 3. Upper Secondary ☐ 4. University

7. Occupation ☐ 1. Agriculture ☐ 2. Temporary employee ☐ 3. Business owner

   ☐ 4. Government employee ☐ 5. State enterprise employee

   ☐ 6. Healthcare worker ☐ 7. monk (years in monkhood)____

   ☐ 8. Others_____

8. Are you aware of your hepatitis C virus status?

   ☐ 1. No ☐ 2. Yes

   If yes, have you ever received treatment? ☐ 1. No ☐ 2. Yes

9. Factor associated with infection

   9.1 Ever received blood transfusion

   ☐ 1. No

   ☐ 2. Yes, Year received_______ (If multiple times, please indicate the first year received)

   9.2 History of illicit drug use via oral route ☐ 1. No ☐ 2. Yes

   9.3 Ever used intravenous illicit drug ☐ 1. No ☐ 2. Yes

   9.4 History of medical surgery ☐ 1. No ☐ 2. Yes

   Type of surgery_______________

Year ________________ (If multiple times, please indicate the first year received)

9.5 Ever received medical treatment via injection by healthcare staff outside of hospital setting

☐ 1. No ☐ 2. Yes

Year ________________ (If multiple times, please indicate the first one received)

9.6 Ever received medical treatment via injection by non-healthcare or unlicensed personnel in the past

☐ 1. No ☐ 2. Yes

Year ________________ (If multiple times, please indicate the first one received)

9.7 Ever receive medical treatment with acupuncture outside of hospital setting

☐ 1. No ☐ 2. Yes

Year________________ (If multiple times, please indicate the first one received)

9.8 Ever been accidentally pricked by injection needles ☐ 1. No ☐ 2. Yes

9.9 Shared shaving razor blades or sharps with others ☐ 1. No ☐ 2. Yes

9.10 Ever had tattoos ☐ 1. No ☐ 2. Yes

9.11 Spouse with HCV ☐ 1. No ☐ 2. Yes

9.12 Family members in the same household with hepatitis disease ☐ 1. No ☐ 2. Yes

If yes, what kind of hepatitis disease?

☐ 1. Chronic hepatitis ☐ 2. Fulminate hepatitis ☐ 3. Cirrhosis ☐ 4. Liver cancer

9.13 History of homosexuality ☐ 1. No ☐ 2. Yes

9.14 History of hemodialysis ☐ 1. No ☐ 2. Yes Year of last dialysis_____

9.15 Ever donated blood ☐ 1. No ☐ 2. Yes Year of last donation_____

9.16 Ever had blood test for hepatitis ☐ 1. No ☐ 2. Yes
