# Supplementary material for: Assessment of hepatitis C virus infection in two adjacent Thai provinces with drastically different seroprevalence
Source: PLoS One. 2017 May 5;12(5):e0177022. doi: 10.1371/journal.pone.0177022 (PMC5419576; doi:10.1371/journal.pone.0177022)
Supplement: S2 File — (PDF) [file pone.0177022.s003.pdf]

Questionnaires (Thai language)

รหัสชื่อ________________________ วันที่เก็บข้อมูล วัน___เดือน______ปี_____

1. อายุ ☐☐ ปี วัน/เดือน/ปี เกิด ___/___/_____

2. เพศ ☐ 1 ชาย ☐ 2 หญิง

3. น้ำหนัก ________กิโลกรัม 4. ส่วนสูง_________เซนติเมตร

5. ภูมิลำเนา จังหวัด _________________อำเภอ____________________ตำบล____________________

6. การศึกษา ☐ 1 ระดับประถม ☐ 2 ระดับมัธยมต้น ☐ 3 ระดับมัธยมปลาย ☐ 4 ระดับมหาวิทยาลัย

7. ท่านทำอาชีพ ☐ 1 เกษตรกรรม ☐ 2 รับจ้าง ☐ 3 เจ้าของกิจการ ☐ 4 ข้าราชการ

   ☐ 5 รัฐวิสาหกิจ ☐ 6 บุคลากรทางการแพทย์ ☐ 7 พระ จำนวนพรรษา_____

   ☐ 8 อื่นๆ_____

8. ท่านทราบว่าติดเชื้อไวรัสตับอักเสบ ซี มาแล้วหรือไม่

   ☐ 1ไม่ทราบ ☐ 2 ทราบ

   8.1 หากทราบแล้ว ท่านเคยได้รับการรักษาหรือไม่ ☐ 1 ไม่เคย ☐ 2 เคย

9. ปัจจัยเสี่ยงที่เกี่ยวข้องกับการติดเชื้อ

   9.1 ท่านมีประวัติได้รับเลือด ☐ 1 ไม่ใช่ ☐ 2 ใช่ เมื่อปี พ.ศ.__________ (ถ้ามีหลายครั้ง กรุณาระบุปี พ.ศ. ครั้งแรกที่ได้รับ)

   9.2 ท่านมีประวัติใช้ยาเสพติดชนิดกิน ☐ 1 ไม่ใช่ ☐ 2 ใช่

   9.3 ท่านเคยใช้เข็มฉีดยาในการเสพยาเสพติด ☐ 1 ไม่เคยใช้ ☐ 2 เคยใช้

   9.4 ท่านมีประวัติได้รับการผ่าตัด ☐ 1 ไม่ใช่ ☐ 2 ใช่ ชนิดการผ่าตัด________________________________ เมื่อปี พ.ศ._______________ (ถ้ามีหลายครั้ง กรุณาระบุปี พ.ศ. ครั้งแรกที่ได้รับการผ่าตัด)

9.5 ท่านเคยได้รับการรักษาพยาบาลด้วยการฉีดยานอกโรงพยาบาลโดยเจ้าหน้าที่สาธารณสุข ☐ 1 ไม่ใช่ ☐ 2 ใช่

เมื่อปี พ.ศ. _______________ (ถ้ามีหลายครั้ง กรุณาระบุปี พ.ศ. ครั้งแรกที่ได้รับการฉีดยาดังกล่าว)

9.6 ท่านเคยรับการรักษาพยาบาลด้วยการฉีดยาจากหมอชาวบ้าน,อสม.,แพทย์ทหารฯลฯในอดีต ☐ 1ไม่ใช่ ☐ 2 ใช่

เมื่อปี พ.ศ_________________(ถ้ามีหลายครั้ง กรุณาระบุปี พ.ศ. ครั้งแรกที่ได้รับการฉีดยาดังกล่าว)

9.7 ท่านเคยได้รับการรักษาพยาบาลด้วยการฝังเข็มนอกโรงพยาบาล ☐ 1 ไม่ใช่ ☐ 2 ใช่

เมื่อปี พ.ศ_________________(ถ้ามีหลายครั้ง กรุณาระบุปี พ.ศ. ครั้งแรกที่ได้รับการฝังเข็ม)

9.8 ท่านมีอุบัติเหตุโดนเข็มฉีดยาตำ ☐ 1 ไม่ใช่ ☐ 2 ใช่

9.9 ท่านใช้มีดโกนและของมีคมร่วมกับผู้อื่น ☐ 1 ไม่ใช่ ☐ 2 ใช่

9.10 ท่านมีประวัติการสัก ☐ 1 ไม่ใช่ ☐ 2 ใช่

9.11 ท่านมีสามีหรือภรรยาที่เป็นโรคไวรัสตับอักเสบ ซี ☐ 1 ไม่ใช่ ☐ 2 ใช่

9.12 ท่านมีบุคคลในครอบครัวที่อาศัยในบ้านเดียวกันเป็นโรคตับ ☐ 1 ไม่ใช่ ☐ 2 ใช่

ถ้าใช่เป็นโรคตับชนิดใด ☐ 1 ตับอักเสบ ☐ 2 ตับวาย ☐ 3 ตับแข็ง ☐ 4 มะเร็งตับ

9.13 ท่านเคยมีประวัติรักร่วมเพศ ☐ 1 ไม่เคย ☐ 2 เคย

9.14 ท่านเคยมีประวัติฟอกเลือด หรือล้างไต ☐ 1 ไม่เคย ☐ 2 เคย ครั้งสุดท้ายเมื่อปี____________

9.15 ท่านเคยบริจาคโลหิต ☐ 1 ไม่เคย ☐ 2 เคย ครั้งสุดท้ายเมื่อปี____________

9.16 ท่านเคยตรวจเลือดพบตับอักเสบ ☐ 1 ไม่เคย ☐ 2 เคย
