# Supplementary material for: Assessment of hepatitis C virus infection in two adjacent Thai provinces with drastically different seroprevalence
Source: PLoS One. 2017 May 5;12(5):e0177022. doi: 10.1371/journal.pone.0177022 (PMC5419576; doi:10.1371/journal.pone.0177022)
Supplement: S1 Table — (DOCX) [file pone.0177022.s004.docx]

**S1 Table.**  **Unassociated factors in univariate analysis with HCV infection in Phetchabun and Khon Kaen.**

|  | **Phetchabun** | | | |  | **Khon Kaen** | | | |
| --- | --- | --- | --- | --- | --- | --- | --- | --- | --- |
| **Parameters** | **All** | **Anti-HCV positive (%)** | **Unadjusted** | |  | **All** | **Anti-HCV positive (%)** | **Unadjusted** | |
|  |  |  | **odds ratio (95% CI)** | ***P* value** |  |  |  | **odds ratio (95% CI)** | ***P* value** |
| **Blood transfusion** | 1623 |  | 1.3 (0.8, 2.1) | 0.276 |  | 1400 |  | 1.4 (0.5, 3.9) | 0.557 |
| No | 1509 | 233 (15.4) |  |  |  | 1317 | 47 (3.6) |  |  |
| Yes | 114 | 22 (19.3) |  |  |  | 83 | 4 (4.8) |  |  |
| **Acupuncture** | 1620 |  | 1.0 (0.4, 2.3) | 0.981 |  | 1396 |  | 2.9 (0.7, 12.9) | 0.159 |
| No | 1575 | 243 (15.4) |  |  |  | 1375 | 48 (3.5) |  |  |
| Yes | 45 | 7 (15.6) |  |  |  | 21 | 2 (9.5) |  |  |
| **Needle stick** | 1643 |  | 0.8 (0.4, 1.6) | 0.500 |  | 1404 |  | 0.9 (0.1, 6.6) | 0.909 |
| No | 1572 | 246 (15.6) |  |  |  | 1372 | 48 (3.5) |  |  |
| Yes | 71 | 9 (12.7) |  |  |  | 32 | 1 (3.1) |  |  |
| **HCV-infected spouse** | 1642 |  | 0.5 (0.3, 1.0) | 0.060 |  | 1395 |  | 1.9 (0.2, 14.7) | 0.539 |
| No | 1540 | 245 (15.9) |  |  |  | 1380 | 50 (3.6) |  |  |
| Yes | 102 | 9 (8.8) |  |  |  | 15 | 1 (6.7) |  |  |
| **Homosexuality** | 1623 |  | 1.4 (0.8, 2.3) | 0.212 |  | 1394 |  | 1.2 (0.2, 9.5) | 0.827 |
| No | 1529 | 235 (15.4) |  |  |  | 1371 | 48 (3.5) |  |  |
| Yes | 94 | 19 (20.2) |  |  |  | 23 | 1 (4.3) |  |  |
