# Supplementary material for: Assessment of hepatitis C virus infection in two adjacent Thai provinces with drastically different seroprevalence
Source: PLoS One. 2017 May 5;12(5):e0177022. doi: 10.1371/journal.pone.0177022 (PMC5419576; doi:10.1371/journal.pone.0177022)
Supplement: S2 Table — (DOCX) [file pone.0177022.s005.docx]

**S2 Table. The estimated number of HCV-seropositive and actively infected individuals in the Lom Kao and Lom Sak districts in Phetchabun and in the Chum Phae district of Khon Kaen.**

| **Phetchabun** | | | | | | | | | | | | | | | | |
| --- | --- | --- | --- | --- | --- | --- | --- | --- | --- | --- | --- | --- | --- | --- | --- | --- |
|  | **Male** | | | | | |  | **Female** | | | | | |  | **Total** | |
| **Age (years)** | **Male population** | **Tested samples** | **Anti-HCV +ve (%)** | **RNA +ve (%)** | **Estimatedanti-HCV carriers** | **Estimated RNA carriers** |  | **Female population** | **Tested samples** | **Anti-HCV +ve (%)** | **RNA +ve (%)** | **Estimated anti-HCV carriers** | **Estimated RNA carriers** |  | **Estimated anti HCV carriers** | **Estimated RNA carriers** |
| **30** | 1457 | 6 | 0 (0.0) | 0 (0.0) | 0 | 0 |  | 1448 | 13 | 0 (0.0) | 0 (0.0) | 0 | 0 |  | 0 | 0 |
| **31** | 1538 | 4 | 0 (0.0) | 0 (0.0) | 0 | 0 |  | 1584 | 5 | 0 (0.0) | 0 (0.0) | 0 | 0 |  | 0 | 0 |
| **32** | 1660 | 11 | 0 (0.0) | 0 (0.0) | 0 | 0 |  | 1579 | 9 | 0 (0.0) | 0 (0.0) | 0 | 0 |  | 0 | 0 |
| **33** | 1671 | 8 | 1 (12.5) | 0 (0.0) | 209 | 0 |  | 1597 | 11 | 1 (9.1) | 1 (100.0) | 145 | 145 |  | 354 | 145 |
| **34** | 1586 | 6 | 0 (0.0) | 0 (0.0) | 0 | 0 |  | 1539 | 11 | 1 (9.1) | 0 (0.0) | 140 | 0 |  | 140 | 0 |
| **35** | 1582 | 9 | 1 (11.1) | 0 (0.0) | 176 | 0 |  | 1658 | 12 | 1 (8.3) | 1 (100.0) | 138 | 138 |  | 313 | 138 |
| **36** | 1770 | 11 | 3 (27.3) | 3 (100.0) | 483 | 483 |  | 1575 | 17 | 2 (11.8) | 2 (100.0) | 186 | 186 |  | 669 | 669 |
| **37** | 1704 | 20 | 5 (25.0) | 4 (80.0) | 426 | 341 |  | 1675 | 22 | 1 (4.5) | 1 (100.0) | 75 | 75 |  | 501 | 416 |
| **38** | 1838 | 22 | 4 (18.2) | 3 (75.0) | 335 | 251 |  | 1807 | 25 | 1 (4.0) | 0 (0.0) | 72 | 0 |  | 407 | 251 |
| **39** | 1839 | 20 | 8 (40.0) | 5 (62.5) | 736 | 460 |  | 1748 | 28 | 2 (7.1) | 1 (50.0) | 124 | 62 |  | 860 | 522 |
| **40** | 1823 | 17 | 3 (17.6) | 2 (66.7) | 321 | 214 |  | 1731 | 33 | 3 (9.1) | 1 (33.3) | 158 | 52 |  | 478 | 266 |
| **41** | 1747 | 24 | 9 (37.5) | 9 (100.0) | 655 | 655 |  | 1730 | 45 | 1 (2.2) | 0 (0.0) | 38 | 0 |  | 693 | 655 |
| **42** | 1667 | 32 | 9 (28.1) | 7 (77.8) | 468 | 364 |  | 1684 | 33 | 2 (6.1) | 1 (50.0) | 103 | 51 |  | 571 | 416 |
| **43** | 1717 | 27 | 6 (22.2) | 5 (83.3) | 381 | 318 |  | 1792 | 26 | 1 (3.8) | 1 (100.0) | 68 | 68 |  | 449 | 386 |
| **44** | 1693 | 29 | 7 (24.1) | 7 (100.0) | 408 | 408 |  | 1629 | 41 | 1 (2.4) | 1 (100.0) | 39 | 39 |  | 447 | 447 |
| **45** | 1698 | 24 | 9 (37.5) | 9 (100.0) | 637 | 637 |  | 1689 | 34 | 1 (2.9) | 1 (100.0) | 49 | 49 |  | 686 | 686 |
| **46** | 1624 | 24 | 5 (20.8) | 4 (80.0) | 338 | 270 |  | 1694 | 40 | 1 (2.5) | 1 (100.0) | 42 | 42 |  | 380 | 313 |
| **47** | 1594 | 26 | 10 (38.5) | 7 (70.0) | 614 | 430 |  | 1690 | 46 | 3 (6.5) | 2 (66.7) | 110 | 73 |  | 724 | 503 |
| **48** | 1549 | 31 | 7 (22.6) | 6 (85.7) | 350 | 300 |  | 1587 | 55 | 3 (5.5) | 1 (33.3) | 87 | 29 |  | 437 | 329 |
| **49** | 1517 | 26 | 13 (50.0) | 12 (92.3) | 759 | 700 |  | 1638 | 39 | 0 (0.0) | 0 (0.0) | 0 | 0 |  | 759 | 700 |
| **50** | 1577 | 42 | 15 (35.7) | 12 (80.0) | 563 | 450 |  | 1702 | 38 | 2 (5.3) | 2 (100.0) | 90 | 90 |  | 653 | 541 |
| **51** | 1568 | 50 | 12 (24.0) | 12 (100.0) | 376 | 376 |  | 1714 | 39 | 1 (2.6) | 0 (0.0) | 45 | 0 |  | 421 | 376 |
| **52** | 1417 | 34 | 12 (35.3) | 9 (75.0) | 500 | 375 |  | 1492 | 25 | 1 (4.0) | 1 (100.0) | 60 | 60 |  | 560 | 435 |
| **53** | 1479 | 30 | 10 (33.3) | 7 (70.0) | 493 | 345 |  | 1523 | 34 | 1 (2.9) | 0 (0.0) | 44 | 0 |  | 537 | 345 |
| **54** | 1458 | 30 | 9 (30.0) | 5 (55.6) | 437 | 243 |  | 1621 | 33 | 2 (6.1) | 1 (50.0) | 99 | 49 |  | 536 | 293 |
| **55** | 1371 | 40 | 13 (32.5) | 11 (84.6) | 446 | 377 |  | 1503 | 32 | 0 (0.0) | 0 (0.0) | 0 | 0 |  | 446 | 377 |
| **56** | 1467 | 31 | 7 (22.6) | 7 (100.0) | 332 | 332 |  | 1580 | 30 | 1 (3.3) | 1 (100.0) | 52 | 52 |  | 384 | 384 |
| **57** | 1266 | 24 | 7 (29.2) | 6 (85.7) | 370 | 317 |  | 1453 | 29 | 1 (3.4) | 0 (0.0) | 49 | 0 |  | 419 | 317 |
| **58** | 1159 | 29 | 11 (37.9) | 11 (100.0) | 439 | 439 |  | 1281 | 20 | 2 (10.0) | 1 (50.0) | 128 | 64 |  | 567 | 503 |
| **59** | 990 | 22 | 5 (22.7) | 3 (60.0) | 225 | 135 |  | 1066 | 21 | 1 (4.8) | 1 (100.0) | 51 | 51 |  | 276 | 186 |
| **60** | 952 | 22 | 5 (22.7) | 2 (40.0) | 216 | 86 |  | 1138 | 21 | 2 (9.5) | 1 (50.0) | 108 | 54 |  | 324 | 140 |
| **61** | 1101 | 15 | 6 (40.0) | 6 (100.0) | 440 | 440 |  | 1219 | 12 | 0 (0.0) | 0 (0.0) | 0 | 0 |  | 440 | 440 |
| **62** | 897 | 16 | 5 (31.3) | 3 (60.0) | 281 | 168 |  | 1024 | 8 | 0 (0.0) | 0 (0.0) | 0 | 0 |  | 281 | 168 |
| **63** | 928 | 3 | 0 (0.0) | 0 (0.0) | 0 | 0 |  | 1041 | 4 | 0 (0.0) | 0 (0.0) | 0 | 0 |  | 0 | 0 |
| **64** | 858 | 9 | 3 (33.3) | 3 (100.0) | 286 | 286 |  | 978 | 2 | 0 (0.0) | 0 (0.0) | 0 | 0 |  | 286 | 286 |
| **Total** | **51762** | **774** | **220 (28.4)** | **180 (23.3)** | **12697** | **10201** |  | **53409** | **893** | **39 (4.4)** | **23 (2.6)** | **2301** | **1432** |  | **14998** | **11633** |

| **Khon Kaen** | | | | | | | | | | | | | | | | |
| --- | --- | --- | --- | --- | --- | --- | --- | --- | --- | --- | --- | --- | --- | --- | --- | --- |
|  | **Male** | | | | | |  | **Female** | | | | | |  | **Total** | |
| **Age (years)** | **Male population** | **Tested samples** | **Anti-HCV +ve (%)** | **RNA +ve (%)** | **Estimated**  **anti-HCV carriers** | **Estimated RNA carriers** |  | **Female population** | **Tested samples** | **Anti-HCV +ve (%)** | **RNA +ve (%)** | **Estimated anti-HCV carriers** | **Estimated RNA carriers** |  | **Estimated anti HCV carriers** | **Estimated RNA carriers** |
| **30** | 644 | 1 | 0 (0.0) | 0 (0.0) | 0 | 0 |  | 566 | 3 | 0 (0.0) | 0 (0.0) | 0 | 0 |  | 0 | 0 |
| **31** | 700 | 4 | 0 (0.0) | 0 (0.0) | 0 | 0 |  | 653 | 6 | 0 (0.0) | 0 (0.0) | 0 | 0 |  | 0 | 0 |
| **32** | 617 | 5 | 0 (0.0) | 0 (0.0) | 0 | 0 |  | 607 | 7 | 0 (0.0) | 0 (0.0) | 0 | 0 |  | 0 | 0 |
| **33** | 626 | 6 | 0 (0.0) | 0 (0.0) | 0 | 0 |  | 618 | 15 | 0 (0.0) | 0 (0.0) | 0 | 0 |  | 0 | 0 |
| **34** | 701 | 7 | 0 (0.0) | 0 (0.0) | 0 | 0 |  | 614 | 11 | 0 (0.0) | 0 (0.0) | 0 | 0 |  | 0 | 0 |
| **35** | 696 | 6 | 0 (0.0) | 0 (0.0) | 0 | 0 |  | 646 | 15 | 1 (6.7) | 1 (100.0) | 43 | 43 |  | 43 | 43 |
| **36** | 664 | 8 | 0 (0.0) | 0 (0.0) | 0 | 0 |  | 634 | 14 | 0 (0.0) | 0 (0.0) | 0 | 0 |  | 0 | 0 |
| **37** | 668 | 8 | 0 (0.0) | 0 (0.0) | 0 | 0 |  | 667 | 16 | 0 (0.0) | 0 (0.0) | 0 | 0 |  | 0 | 0 |
| **38** | 628 | 7 | 0 (0.0) | 0 (0.0) | 0 | 0 |  | 673 | 18 | 1 (5.6) | 0 (0.0) | 38 | 0 |  | 38 | 0 |
| **39** | 672 | 12 | 0 (0.0) | 0 (0.0) | 0 | 0 |  | 648 | 23 | 1 (4.3) | 0 (0.0) | 28 | 0 |  | 28 | 0 |
| **40** | 639 | 9 | 0 (0.0) | 0 (0.0) | 0 | 0 |  | 632 | 26 | 0 (0.0) | 0 (0.0) | 0 | 0 |  | 0 | 0 |
| **41** | 694 | 19 | 2 (10.5) | 2 (100.0) | 73 | 73 |  | 716 | 27 | 0 (0.0) | 0 (0.0) | 0 | 0 |  | 73 | 73 |
| **42** | 596 | 8 | 2 (25) | 2 (100.0) | 149 | 149 |  | 657 | 18 | 0 (0.0) | 0 (0.0) | 0 | 0 |  | 149 | 149 |
| **43** | 741 | 22 | 2 (9.1) | 1 (50.0) | 67 | 34 |  | 703 | 32 | 1 (3.1) | 1 (100.0) | 22 | 22 |  | 89 | 56 |
| **44** | 717 | 16 | 3 (18.8) | 3 (100.0) | 135 | 135 |  | 710 | 33 | 0 (0.0) | 0 (0.0) | 0 | 0 |  | 135 | 135 |
| **45** | 689 | 28 | 4 (14.3) | 3 (75.0) | 99 | 74 |  | 735 | 42 | 0 (0.0) | 0 (0.0) | 0 | 0 |  | 99 | 74 |
| **46** | 647 | 19 | 1 (5.3) | 1 (100.0) | 34 | 34 |  | 698 | 47 | 0 (0.0) | 0 (0.0) | 0 | 0 |  | 34 | 34 |
| **47** | 705 | 18 | 0 (0.0) | 0 (0.0) | 0 | 0 |  | 739 | 37 | 0 (0.0) | 0 (0.0) | 0 | 0 |  | 0 | 0 |
| **48** | 688 | 20 | 2 (10) | 1 (50.0) | 69 | 35 |  | 724 | 42 | 0 (0.0) | 0 (0.0) | 0 | 0 |  | 69 | 35 |
| **49** | 648 | 23 | 3 (13.0) | 1 (33.3) | 84 | 28 |  | 695 | 32 | 0 (0.0) | 0 (0.0) | 0 | 0 |  | 84 | 28 |
| **50** | 639 | 23 | 0 (0.0) | 0 (0.0) | 0 | 0 |  | 679 | 36 | 1 (2.8) | 0 (0.0) | 19 | 0 |  | 19 | 0 |
| **51** | 694 | 29 | 4 (13.8) | 2 (50.0) | 96 | 48 |  | 704 | 46 | 0 (0.0) | 0 (0.0) | 0 | 0 |  | 96 | 48 |
| **52** | 570 | 27 | 3 (11.1) | 1 (33.3) | 63 | 21 |  | 572 | 47 | 1 (2.1) | 1 (100.0) | 12 | 12 |  | 75 | 33 |
| **53** | 528 | 22 | 0 (0.0) | 0 (0.0) | 0 | 0 |  | 585 | 39 | 0 (0.0) | 0 (0.0) | 0 | 0 |  | 0 | 0 |
| **54** | 541 | 24 | 1 (4.2) | 1 (100.0) | 23 | 23 |  | 536 | 31 | 0 (0.0) | 0 (0.0) | 0 | 0 |  | 23 | 23 |
| **55** | 587 | 26 | 2 (7.7) | 0 (0.0) | 45 | 0 |  | 625 | 36 | 0 (0.0) | 0 (0.0) | 0 | 0 |  | 45 | 0 |
| **56** | 522 | 31 | 4 (12.9) | 2 (50.0) | 67 | 34 |  | 553 | 20 | 0 (0.0) | 0 (0.0) | 0 | 0 |  | 67 | 34 |
| **57** | 497 | 21 | 1 (4.8) | 1 (100.0) | 24 | 24 |  | 516 | 29 | 2 (6.9) | 1 (50.0) | 36 | 18 |  | 60 | 42 |
| **58** | 451 | 22 | 1 (4.5) | 1 (100.0) | 20 | 20 |  | 480 | 23 | 0 (0.0) | 0 (0.0) | 0 | 0 |  | 20 | 20 |
| **59** | 425 | 25 | 2 (8.0) | 2 (100.0) | 34 | 34 |  | 443 | 20 | 1 (5.0) | 1 (100) | 22 | 22 |  | 56 | 56 |
| **60** | 361 | 19 | 2 (10.5) | 1 (50.0) | 38 | 19 |  | 399 | 19 | 0 (0.0) | 0 (0.0) | 0 | 0 |  | 38 | 19 |
| **61** | 436 | 17 | 1 (5.9) | 0 (0.0) | 26 | 0 |  | 444 | 12 | 0 (0.0) | 0 (0.0) | 0 | 0 |  | 26 | 0 |
| **62** | 391 | 14 | 1 (7.1) | 1 (100.0) | 28 | 28 |  | 428 | 11 | 0 (0.0) | 0 (0.0) | 0 | 0 |  | 28 | 28 |
| **63** | 381 | 10 | 1 (10.0) | 0 (0.0) | 38 | 0 |  | 391 | 13 | 0 (0.0) | 0 (0.0) | 0 | 0 |  | 38 | 0 |
| **64** | 341 | 0 | 0 (0.0) | 0 (0.0) | 0 | 0 |  | 384 | 8 | 0 (0.0) | 0 (0.0) | 0 | 0 |  | 0 | 0 |
| **Total** | **20744** | **556** | **42 (7.5)** | **26 (4.7)** | **1212** | **813** |  | **21074** | **854** | **9 (1.0)** | **5 (0.6)** | **220** | **117** |  | **1432** | **930** |
