# Supplementary material for: Assessment of hepatitis C virus infection in two adjacent Thai provinces with drastically different seroprevalence
Source: PLoS One. 2017 May 5;12(5):e0177022. doi: 10.1371/journal.pone.0177022 (PMC5419576; doi:10.1371/journal.pone.0177022)
Supplement: S3 Table — (DOCX) [file pone.0177022.s006.docx]

**S3 Table. HCV seroprevalence in Chum Phae district in Khon Kaen in 2014 [9] and this study.**

| **Age group (years)** | **Year 2014** | |  | **Year 2015** | |
| --- | --- | --- | --- | --- | --- |
|  | **Sample**  **number** | **Anti-HCV positive (%)** |  | **Sample**  **number** | **Anti-HCV positive (%)** |
| 30-39 | 190 | 2 (1.0) |  | 192 | 3 (1.6) |
| 40-49 | 185 | 11 (5.9) |  | 518 | 20 (3.9) |
| 50-59 | 205 | 5 (2.4) |  | 577 | 23 (4.0) |
| 60-64 | 12 | 0 (0.00) |  | 123 | 5 (4.1) |
| **TOTAL** | **592** | **18 (3.0)** |  | **1410** | **51 (3.6)** |
